# Magnesium isoglycyrrhizinate suppresses bladder cancer progression by modulating the miR-26b/Nox4 axis

**DOI:** 10.1080/21655979.2022.2031677

**Published:** 2022-03-16

**Authors:** Zhihao Yuan, Guancheng Guo, Guifang Sun, Qi Li, Lihui Wang, Baoping Qiao

**Affiliations:** a Department of Emergency, The First Affiliated Hospital of Zhengzhou University, Zhengzhou, P. R. China; b Department of Neurology, The First Affiliated Hospital of Zhengzhou University, Zhengzhou, P. R. China; c Department of Urology, The First Affiliated Hospital of Zhengzhou University, Zhengzhou, P. R. China

**Keywords:** Magnesium isoglycyrrhizinate, bladder cancer, miR-26b, Nox4/ NF-κB/HIF-1α, apoptosis

## Abstract

Magnesium isoglycyrrhizinate (MI), a magnesium salt of 18α-GA stereoisomer, has been reported to exert efficient hepatoprotective activity. However, its effect on bladder cancer remains unclear. The study explored the effects of MI on the growth, colony formation, apoptosis, invasion, and migration of bladder cancer cells (HTB9 and BIU87 cells). Typical apoptotic changes of bladder cancer cells such as nuclear concentration and fragmentation were observed using Hoechst staining. The effects of MI on the expression levels of microRNA-26b (miR-26b), NADPH oxidase 4 (Nox4), nuclear transcription factor-κB (NF-κB), and hHypoxia inducible factor-1α (HIF-1α) were detected using qRT-PCR and Western blot. The potential targets of miR-26b were predicted using Targetscan, and their interactions were determined by luciferase reporter assay. A xenograft mouse model was established to evaluate the anti-tumor effects of MI *in vivo*. MI significantly suppressed the proliferation, colony formation, invasion, and migration and induced apoptosis of human bladder cancer cells, and MI significantly increased miR-26b expression. Nox 4 was identified to be a direct target of miR-26b. MiR-26b mimics significantly decreased the relative luciferase activity of wild type (WT) Nox 4 but not mutant type (MUT) Nox4. Meanwhile, MI markedly downregulated the expression levels of Nox4, NF-κB, and HIF-1α both *in vitro* and *in vivo*. Moreover, MI inhibited xenograft tumor growth *in vivo* and decreased the expression of Nox4, NF-κB, and HIF-1α. Overall, MI showed a potent anti-tumor effect against bladder cancer partially via modulating the miR-26b/Nox4 axis.

## Graphical abstract

**Figure:**
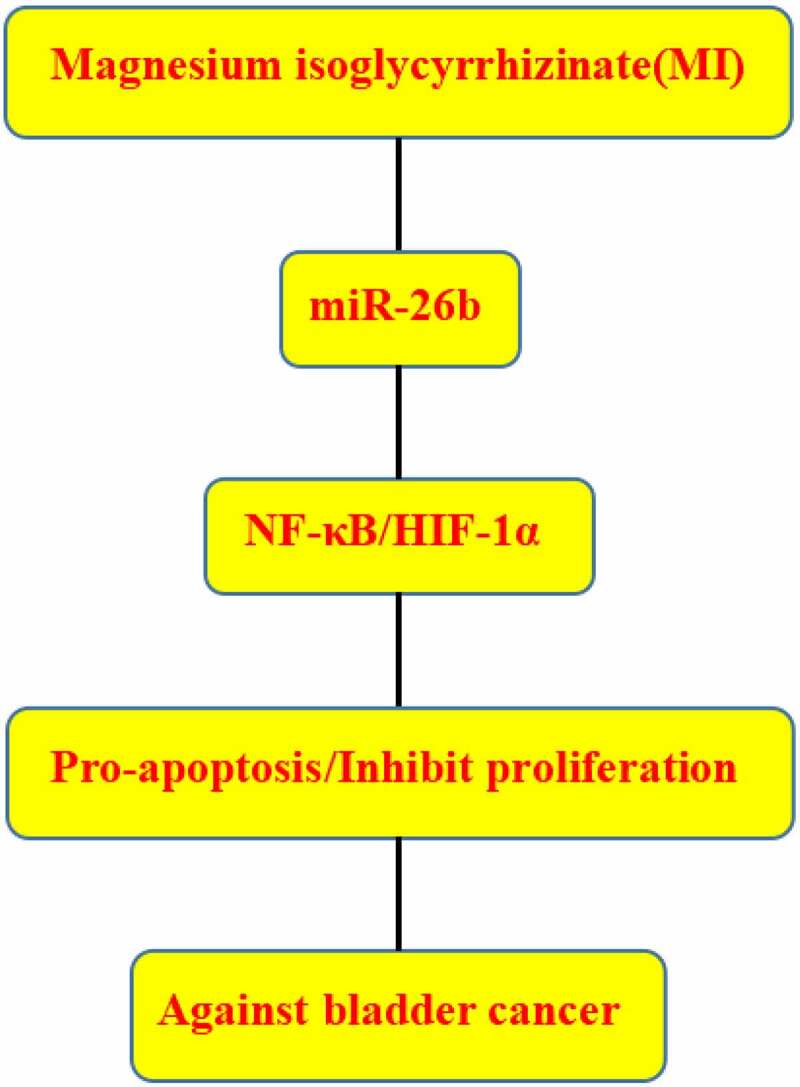

## Background

Bladder cancer has become a common malignancy with higher morbidity and mortality and an estimated 430,000 newly diagnosed cases every year worldwide [1,2]. The incidence of bladder cancer is higher in men, suggesting it is primarily an environmental disease with geographic variation [3]. Even worse, approximately one‐third of patients with muscle‐invasive bladder cancer have undetected metastases at the time of treatment [4]. Although considerable progress has been achieved in surgical techniques and adjuvant chemotherapies, bladder cancer mortality is still high [5]. Many studies have demonstrated that the occurrence and development of bladder cancer are complex processes with abnormal genetic changes or epigenetic abnormalities [4–6]. Therefore, a better understanding of the specific mechanisms underlying bladder cancer progression will contribute to identifying efficient drug targets and even expand the treatment window period in bladder cancer.

Magnesium isoglycyrrhizinate (MI), a magnesium salt of 18α-GA stereoisomer, is an efficient hepatoprotective agent [7–9]. Expect its protective effect on the liver, MI exerts a potential protective effect in various human diseases. For instance, MI can prevent drug-induced liver damages after the initial chemotherapy for patients with early-stage gastrointestinal cancer [10]. One previous study reported that liver toxicities induced by paclitaxel plus cisplatin chemotherapy could potentially decrease the ability of hepatic elimination and increase system exposure of paclitaxel, while MI efficiently helps restore hepatic clearance of paclitaxel [11]. MI can protect against renal‑ischemia‑reperfusion injury in a mouse model via its antiinflammation, anti‑oxidation, and anti‑apoptosis capacities [12]. MI has been demonstrated to inhibit myocardial hypertrophy by inactivating the TLR4/NF-κB signaling pathway in mice [13]. In addition, MI also ameliorates fructose-induced podocyte apoptosis by downregulating miR-193a to upregulate Wilms’ tumor protein (WT1) expression [14]. However, the effect of MI on bladder cancer has not been well studied.

NADPH oxidase (Nox4), which can generate H_2_O_2_ reactive oxygen species, is highly expressed in human tumors [15]. NOX4 can support glycolysis and promote glutamine metabolism in non-small cell lung cancer (NSCLC) cells [16] and drive ROS formation via GLI1 pathway to regulate gastric cancer cell proliferation and apoptosis [17]. Nox4 is closely correlated with gastric cancer progression and predicts a poor prognosis [18]. It has been reported that Nox4 inhibition might alleviate ROS generation and prevent NF-κB activation and its translocation into the nucleus [19]. In addition, recent studies have revealed that controlling HIF-1α gene by NF-κB is crucial under hypoxic stress [20,21]. Although Nox4 also plays an essential role in bladder cancer development [22], the regulatory axis of Nox4 in bladder cancer has not been well studied.

MicroRNAs (miRNAs), a group of small non-coding RNAs with approximately 22 nucleotides in length, often regulate gene expression post-transcriptionally through binding to the 3’-untranslated region (3’-UTR) of the target mRNAs [23]. Many miRNAs are significantly involved in bladder cancer progression. Besides miR‑223 [24], miR-1265 [25], miR-125a-5p [26], and miR-155 [27], miR-217 inhibits bladder cancer cell proliferation and migration through targeting KMT2D [28]. MiR-124-3p suppresses bladder cancer cell migration and invasion through targeting ITGA3 [29]. MiR-129-5p suppresses gemcitabine resistance and promotes apoptosis of bladder cancer cells via targeting Wnt5a [30]. We have previously reported that miR-26b acts as a tumor suppressor in bladder cancer [31]. In this study, we explored the effects of MI on bladder cancer cell growth, colony formation, apoptosis, invasion, and migration *in vitro* and the underlying molecular mechanisms for the first time. We found that MI efficiently inhibits bladder cancer progression both *in vitro* and *in vivo* by deactivating the Nox 4/NF-κB/HIF-1α signaling pathway via upregulating miR-26b expression. Our results provide new insight into MI function in bladder cancer and indicate that MI might be a potential anti-tumor agent against bladder cancer.

## Methods

### Materials

**Figure 1.**
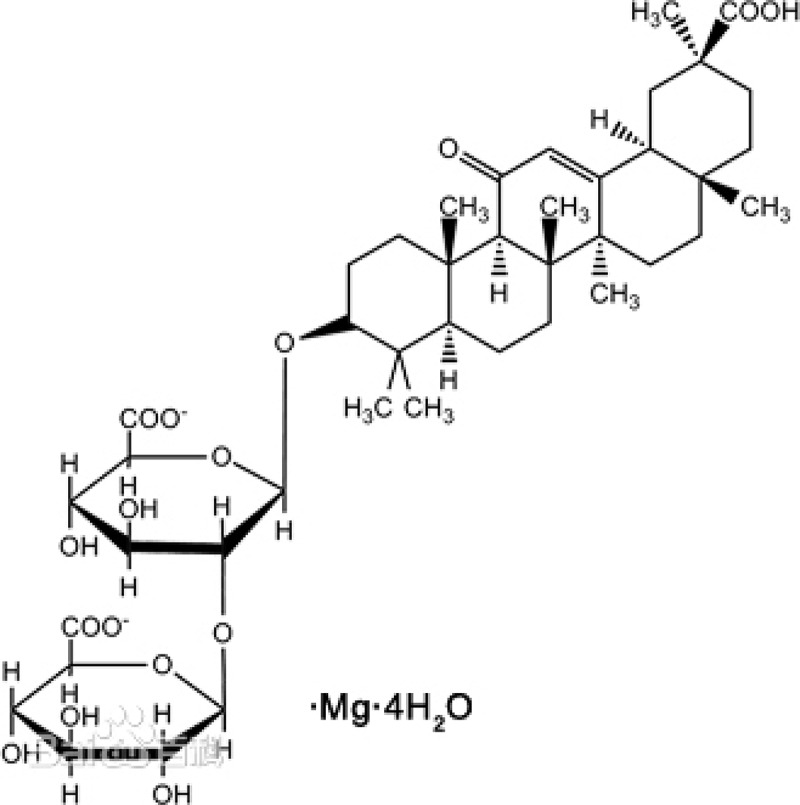
The structural of MI.

Magnesium isoglycyrrhizinate (MI) (purity > 98%, monohydrate) was obtained from Zhengda Tianqing Pharmaceutical Co., Ltd (Jiangsu, China). All other chemicals and solvents were commercially available. Figure 1 shows the structure of MI.

### Cell culture

Normal urothelial cell-line SV-HUC-1 (C1299) and human bladder cancer cells HTB9 (C5637) and BIU87 (C3003) were purchased from Cell Bank of Chinese Academy of Sciences (Shanghai, China) and cultured in RPMI-1640 media (Solarbio, Beijing, China) containing 10% FBS (Gibco BRL, Grand Island, NY, USA), 100 unit/mL penicillin, and 0.1 mg/mL streptomycin at 37°C with 5% CO_2_. The number of samples used for each group was 6.

### Cell transfection

Cell transfection was performed using Lipofectamine 2000 (Invitrogen) according to the manufacturer’s instructions. MiR-26b mimics (HMI0419) 5’- UUCAAGUAAUUCAGGAUAGGU -3’, miR-26b inhibitor (HLTUD0420) 5’-ACCUAUCCUGAAUUACUUGAA -3’, the corresponding negative control miR-NC(HLTUD002):5’- UUCUCCGAACGUGUCACGUTT -3’, and inhibitor NC (HLTUD002C) 5’-ACGUGACACGUUCGGAGAATT -3’) were purchased from Sigma Aldrich (Shanghai, China).

### Luciferase reporter assay

The binding site of miR-26b to the 3′-UTR of Nox4 was predicted using a bioinformatics tool TargetScan. To confirm their interaction, wild-type or mutant-binding sequences of miR-26b in Nox4 3′-UTR were synthesized and sub-cloned into pmirGLO dual-luciferase vector (Promega, Madison, WI, USA). Nox4-WT/Mut vector (Supplementary table S1 and table S2) was co-transfected with miR-26b mimics, NC mimics, miR-26b inhibitor or NC inhibitor into HTB9 or BIU87 cells. 48 h later, luciferase activity was monitored using Dual Luciferase Report Assay System (Dual-Glo, Promega, USA).

### MTT assay

Cell viability was evaluated using MTT assay as previously described [32]. Briefly, logarithmic phase SV-HUC-1, HTB9 or BIU87 cells were collected, resuspended as 2 × 10^5^ cells/mL suspension, and cultured in 96-well plates at 37°C in an incubator with 5% CO_2_. After treated with MI at 0, 1. 2, 5, 10, and 20 mg/mL for 24 h, cells were incubated with 10 μL MTT solution (0.5 mg/mL, Sigma) for another 5 h. After the medium was aspirated, the formed formazan crystals were dissolved in 150 μL DMSO (Sigma), and the absorbance at 630 nm was detected using a microplate reader (Biotek, SYNERGY HTX, VT, USA).

### Colony formation assay

Colony formation assay was performed as previously described [6]. In brief, HTB9 and BIU87 cells were seeded into 6-well plates at a density of 1,000 cells per well and cultured in media with or without 3.82 or 2.85 mg/mL MI for two weeks. After washed with PBS twice, cells were fixed with methanol and stained with 0.1% crystal violet for 25 min. The colonies were imaged and counted using a light microscope (CKX31, Olympus, Tokyo, Japan).

### Hoechst 33,258 staining assay

The changes in cellular morphology of HTB9 and BIU87 cells were observed using Hoechst 33,258 staining (Sigma), as previously described [33]. Briefly, HTB9 and BIU87 cells were seeded into 6-well plates and cultured in media with or without 3.82 or 2.85 mg/mL MI overnight. Then, cells were fixed with 4% formaldehyde for 15 min and stained with Hoechst 33,258 (10 mg/L) for another 1 h. After washing with PBS twice, cells were subjected to fluorescence microscopy (DP71, Olympus, Tokyo, Japan). Meanwhile, morphological changes, including volume reduction and nuclear chromatin condensation, were observed.

### Transwell assay

The invasion and migration capacities of HTB9 and BIU87 cells were evaluated by Transwell assays using 24-well Transwell™ plates (3422, Lumiprobe, Shanghai, China) coated with or without Matrigel (356,234, Lumiprobe, Shang Hai, China), as previously described [34]. Briefly, approximately 1 × 10^5^ cells in serum-free medium were seeded into the upper chamber, and the lower chamber was filled with media containing 20% FBS. The migration assay was performed similarly without coating the membranes with Matrigel™. After 24 h of incubation, cells were fixed with 4% paraformaldehyde and stained with 0.1% crystal violet. Finally, the numbers of invaded and migrated cells were counted in five randomly selected fields under a light microscope.

### qRT-PCR

Total RNAs were extracted from cultured cells using TRIzol reagent (Invitrogen) according to the manufacturer’s instructions. Approximately 1.2 μg RNA was reversely transcribed into cDNA using the Prime Script RT Master Mix (TaKaRa, Japan), and the qRT-PCR reactions were performed using the SYBR Premix Ex Taq II kit (Takara, Otsu, Japan) based on a Quantstudio™ DX system (Applied Biosystems, Singapore) at conditions of 5 min at 95°C followed by 40 cycles of 95°C for 30 s and 65°C for 45 s. The relative expression changes of targets were analyzed by the 2^−∆∆Ct^ method [35]. GAPDH and U6 were considered as the internal reference for mRNA and miRNA, respectively. The primers were miRNA-26b forward 5′-CTGATGGTTAAGAGAATGT-3′ and reverse 5′-GTCCTTGGACATCCGGGCCG-3′; Nox4 forward 5′-GATGTTGGGGCTAGGATTGT-3′ and reverse 5′-TCTGTGATCCTCGGAGGTAA-3′; NF-kB forward 5′-CACTTATGGACAACTATGAGGT

CTCTGG-3′ and reverse 5′-CTGTCTTGTGGACAACGCAGTGGAATTTTAGG-3′; HIF-1α forward 5′-AAGAACCTGCCCCAGAAATCACCTGTCTC-3′ and reverse 5′-AATGATTTCTGGGGCAGGTTCCCTGTCTC-3′; GAPDH forward 5′-CGAGAG

AATCCGCGGACAT-3′ and reverse 5′-TTGTGCAATACAGCGTGGAC-3′; and U6 forward 5′-GACAGATTCGGTCTGTGGCAC-3′ and reverse 5′-GATTACCCGTCG

GCCATCGATC-3′. All primers were designed using primer 5.0 based on the cDNA sequence, which was downloaded from NCBI (https://www.ncbi.nlm.nih.gov/).

### Western blot

Total proteins were isolated from cultured cells using RIPA Lysis Buffer (Beyotime, Beijing, China) according to the manufacturer’s instructions and quantified using the BCA method (P0012S, Beyotime, Beijing, China). Approximately equal amounts of proteins in 20–40 μL were separated by 12% SDS-PAGE and transferred onto PVDF membranes. After blocked with 5% nonfat milk, the membranes were incubated first with primary antibodies against Nox4 (Ab9574, 1:1000, Abcam), NF-κB p65 (Ab3771,1:1000, Abcam), HIF-1α (Ab9574, 1:1000, Abcam), and β-actin (Ab8227, 1:1000, Abcam, Cambridge, MA) at 4°C overnight and then with appropriate HRP-conjugated secondary antibodies at room temperature for another 1 h. Positive signals were detected using an ECL kit (Millipore, Germany), and the bands were visualized using a Bio-Rad imaging system and quantitatively analyzed using Image J software.

### Flow cytometer assay

Cell apoptosis was evaluated using the Annexin V-FITC/PI apoptosis detection kit (Keygen Biotech, Nanjing, China) according to the manufacturer’s instructions. Briefly, cells treated with or without 3.82 or 2.85 mg/mL MI were seeded into 6-well plates at a density of approximately 1 × 10^5^ cells/mL. The cells were collected, harvested, washed, and re-suspended in the binding buffer. After stained with Annexin V-FITC at room temperature for 20 min in the dark, cells were incubated with PI for another 10 min. The apoptosis rate of cells was analyzed using a FACSCanton^TM^II flow cytometer (FACSCanton^TM^II, BD, Shanghai, China).

### Animal model

The female BALB/c nude mice (6-week-old, weighing approximately 20.0 ± 2.0 g) were purchased from the First Affiliated Hospital of Zhengzhou University. All mice were kept at room temperature with 12/12-h light-dark cycle and handled strictly according to the Legislation of the Use and Care of Laboratory Animals of China. This study was approved by the Ethics Committee of The First Affiliated Hospital of Zhengzhou University. Because MI’s inhibitory effects on HTB9 cells were better than other cell lines, HTB9 cells were used to further explore the effects of MI on bladder cancer mice. HTB9 tumor-bearing mouse model was established as previously described with minor modification [36]. In brief, HTB9 cells were intraperitoneally injected into the abdominal cavity of BALB/c nude mice, and the ascites were removed from mice and diluted to 1 × 10^7^ cells/mL with physiological saline. Then, 0.2 mL of cells were subcutaneously injected into the right armpit of BALB/c nude mice to establish the solid tumor model. To determine the effects of MI *in vivo*, mice were randomly divided into model group and MI group, with 10 mice in each group. Mice in the MI groups were intragastrically given MI at the dose of 0.1 mL/10 g body weight once per day for 5 weeks at 9:00 am, and mice in the model groups were given the same volume of normal saline simultaneously. Tumor volume was calculated as length× width [2]/2. When experiments were finished, mice were sacrificed, and the excised tumors were removed, weighed, and photographed. Tumor sections were subjected to Ki-67 staining assay using the Ki-67 kit from 4A Biotech (EB06989, Beijing, China).

## Statistical analysis

All data were presented as the mean ± standard error (SE). The differences between groups and among multiple groups were determined by two-tailed Student’s t-test and one-way ANOVA, respectively, using the SPSS software v18.0. P < 0.05 as considered statistically significant.

## Results

A series of *in vitro* and *in vivo* experiments were conducted to explore the effects of MI on bladder cancer. The results showed that MI significantly decreased the expression of Nox4, which was identified to be a direct target miR-26b. Moreover, MI markedly downregulated NF-κB and HIF-1α expression, suppressed bladder cancer cell proliferation, and induced bladder cancer cell apoptosis, thereby inhibiting bladder cancer development.

### *MI inhibited the growth and colony formation of bladder cancer cells* in vitro

**Figure 2.**
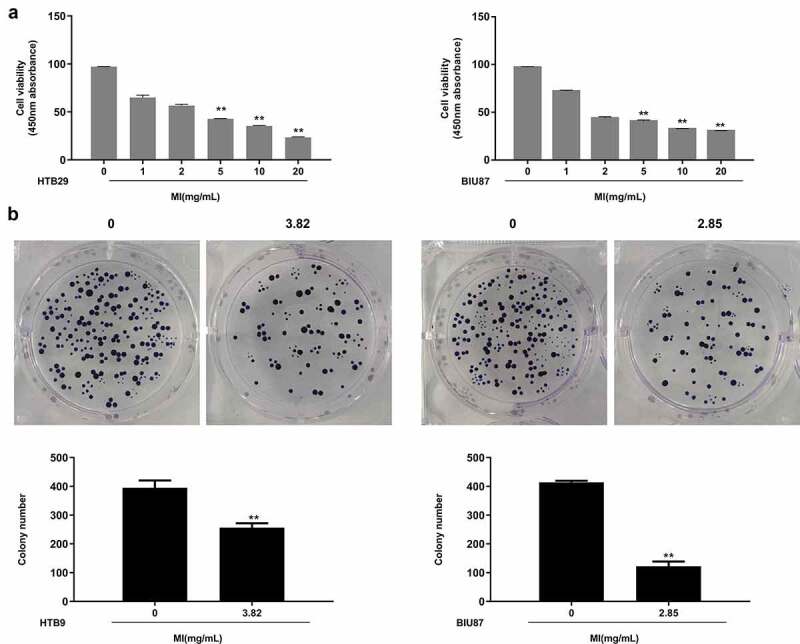
**MI inhibited the growth and colony formation of bladder cancer cells *in vitro***. (a) SV-HUC-1, HTB9, and BIU87 cells were treated with 0, 1. 2, 5, 10, and 20 mg/mL MI for 24 h, and cell viability was evaluated by MTT assay. (b) HTB9 cells were treated with 3.82 mg/mL MI, and BIU87 cells were treated with 2.85 mg/mL MI, and the cell growth was evaluated by colony formation assay. ** P < 0.01.

To explore the effects of MI on bladder cancer, normal urothelial cells SV-HUC-1 and bladder cancer cells HTB9 and BIU87 were treated with MI at 0, 1, 2, 5, 10, and 20 mg/mL for 24 h. MTT assay indicated that MI did not significantly affect the viability of SV-HUC-1 cells but obviously decreased the viability of HTB9 and BIU87 cells in a dose-dependent manner, showing significant differences at 5, 10, and 20 mg/mL in both (p < 0.01), indicating that MI had selective toxicity toward different bladder cancer cells (Figure 2a). Further, the IC_50_ value calculated using SPSS software was 3.82 mg/mL in HTB9 cells and 2.85 mg/mL in BIU87 cells, which were selected for the subsequent experiments. Then, the colony formation assay was performed, and the results showed that MI significantly inhibited the colony formation of both HTB9 (p < 0.01) and BIU87 cells (p < 0.01) (Figure 2b). These results suggested that MI could efficiently inhibit the growth of bladder cancer cells and had high selectivity to tumor cells *in vitro*.

### *MI promoted the apoptosis of bladder cancer cells* in vitro

**Figure 3.**
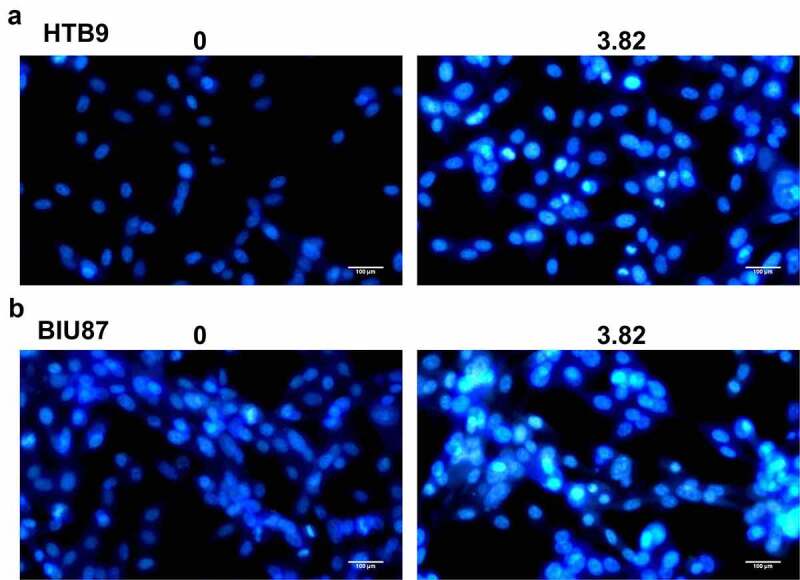
**Effect of MI on the morphological changes in bladder cancer cells was observed by Hoechst 33,258 staining assay**. (a) the morphological changes in bladder cancer HTB9 cells were observed by Hoechst 33,258 staining assay; (b) the morphological changes in bladder cancer BIU87 cells were observed by Hoechst 33,258 staining assay. Magnification, × 400. Scale bar = 50 μm.

Further, the apoptosis of bladder cancer cells HTB9 and BIU87 was observed using Hoechst 33,258 staining assay (Figure 3). MI exposure significantly increased apoptotic morphological changes, including chromosome condensation and nuclear fragmentation, in both HTB9 (Figure 3a) and BIU87 cells (Figure 3b), with the ratio of apoptotic cells between 20% and 30%. These results suggested that MI significantly promoted chromosome condensation and nuclear fragmentation in bladder cancer cells *in vitro*.

### *MI inhibited the invasion and migration and induced apoptosis of bladder cancer cells* in vitro

**Figure 4.**
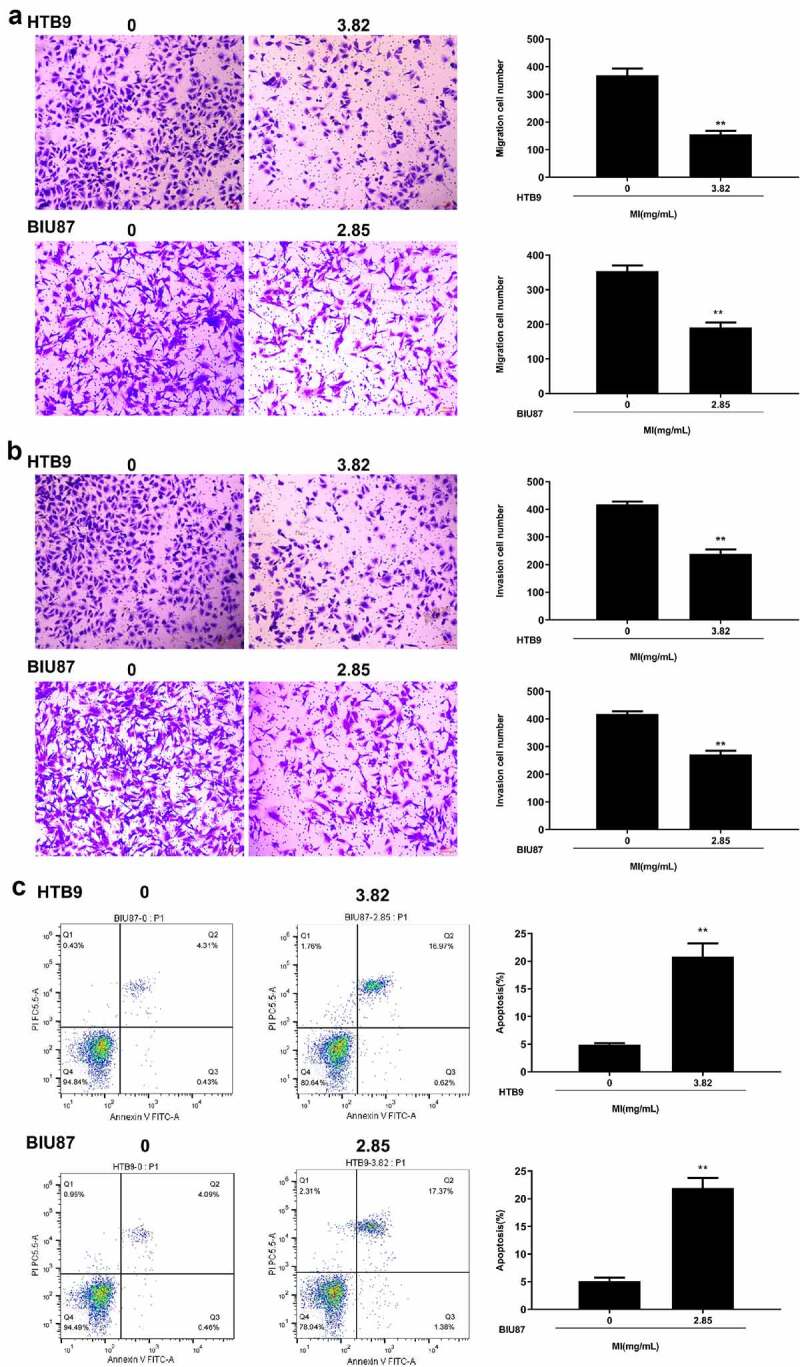
**Effect of MI on invasion, migration, and apoptosis of bladder cancer cells *in vitro***. HTB9 were treated with 3.82 mg/mL MI, and BIU87 cells were treated with 2.85 mg/mL MI. (a and b) The migration (a) and invasion (b) capacities of HTB9 and BIU87 cells were evaluated by Transwell assays. Magnification, × 200, scale bar = 100 μm. (c) The apoptosis rate of HTB9 and BIU87 cells was evaluated by flow cytometry using both forward scatter (FSC) and side scatter (SSC) gating, and the cell population to be gated is set according to the distribution of different cells on the FSC *vs*. SSC scatter plot. ** P < 0.01.

Next, Transwell assay was performed to explore the effect of MI on cell invasion and migration. The results indicated that MI significantly inhibited the migration capacity of both HTB9 (p < 0.01) and BIU87 cells (p < 0.01) (Figure 4a) and markedly suppressed the invasion capacity of both HTB9 (p < 0.01) and BIU87 cells (p < 0.01) (Figure 4b). Meanwhile, the effect of MI on the apoptosis rate of HTB9 and BIU87 cells was evaluated by flow cytometry, and the results revealed that MI significantly promoted the apoptosis of both HTB9 (p < 0.01) and BIU87 cells (p < 0.01) (Figure 4c). These results suggested that MI efficiently inhibited the invasion and migration and induced apoptosis of bladder cancer cells *in vitro*.

### *MI suppressed the activation of Nox4/NF-κB/HIF-1α signaling pathways* in vitro

**Figure 5.**
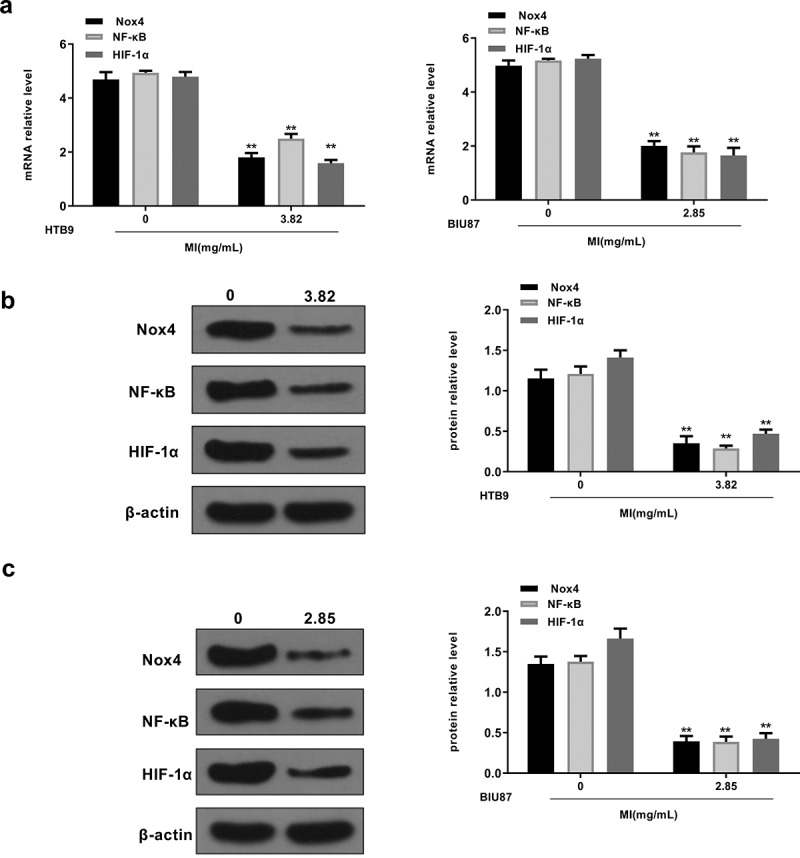
**MI inhibited Nox4/NF-κB/HIF-1α signaling activation *in vitro***. HTB9 were treated with 3.82 mg/mL MI and BIU87 cells were treated with 2.85 mg/mL MI. (a) Nox4, NF-κB, and HIF-1α mRNA levels were evaluated by qRT-PCR. (b and c) Nox4, NF-κB, and HIF-1α protein levels in BTB9 (b) and BIU87 cells (c) were evaluated by Western blot. ** P < 0.01. Full-length blots/gels are presented in Supplementary Figs. 1 and 2.

We also explored the effects of MI on the Nox4/NF-κB/HIF-1α signaling pathway *in vitro*. HTB9 cells were treated with 3.82 mg/mL MI and BIU87 cells were treated with 2.85 mg/mL MI, and the expression levels of Nox4, NF-κB, and HIF-1α were evaluated by qRT-PCR and Western blot. The results showed that MI significantly decreased mRNA levels of Nox4 (p < 0.01), NF-κB (p < 0.01), and HIF-1α (p < 0.01) in both HTB9 and BIU87 cells (Figure 5a). In addition, MI obviously decreased the protein levels of Nox4, NF-κB (p < 0.01), and HIF-1α in HTB9 cells (p < 0.01 for all, Figure 5b) and in BIU87 cells (p < 0.01 for all, Figure 5c). These results indicated that MI inhibited Nox4/NF-κB/HIF-1α signaling pathway activation in bladder cancer cells *in vitro*.

### Nox4 was a direct target of miR-26b

**Figure 6.**
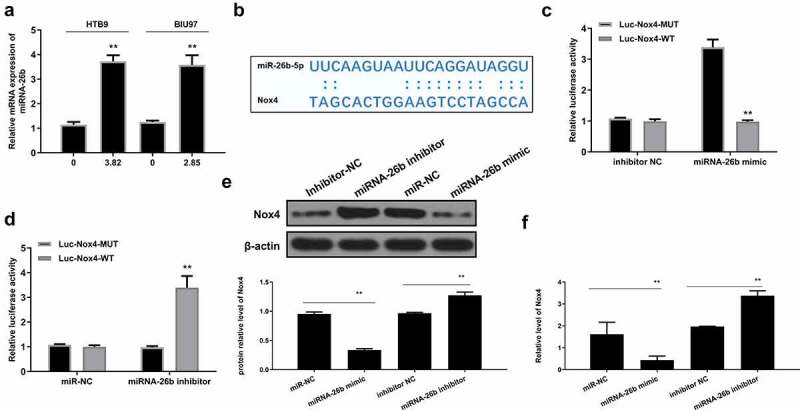
**Nox4 was a target of miR-26b**. (a) HTB9 were treated with 3.82 mg/mL MI and BIU87 cells were treated with 2.85 mg/mL MI. MiR-26b mRNA level was evaluated by qRT-PCR. (b) The interaction between miR-26b and Nox4 was predicted by Targetscan. (c) HTB9 cells were co-transfected with luciferase reporte*r plasmid*s containing WT or MUT Nox4 and miR-26b mimics/miR/NC, or miR-26b inhibitor/inhibitor NC, and the relative luciferase activity was detected by dual luciferase reporter system. (d-e) HTB9 cells were co-transfected with miR-26b mimics, miR-NC, miR-26 inhibitor, inhibitor NC, and Nox4 protein and mRNA levels were evaluated by Western blot and qRT-PCR, respectively. ** P < 0.01. Full-length blots/gels are presented in supplementary Fig. 3.

Interestingly, we found that MI significantly increased miR-26b expression in both HTB9 (p < 0.01) and BIU87 cells (p < 0.01) (Figure 6a). The potential-binding site of miR-26b on the 3’-UTR of Nox4 was predicted using Starbase 2.0 software (http://starbase.sysu.edu.cn/starbase2/index.php) and TargetScan (www.targetscan.org/vert_71/) database (Figure 6b). The results revealed a putative binding site between miR-26b and Nox4, suggesting that Nox4 might be a target of miR-26b. Then, luciferase reporter assay was performed and revealed that miR-26b mimics impaired the luciferase activity of Nox4-Wt (p < 0.01) but not Nox4-Mut. Meanwhile, miR-26b inhibitor significantly increased the relative luciferase activity of Nox4-Wt vector compared with the inhibitor NC inhibitor group (p < 0.01) but had no obvious impact on the Nox4-Mut group in HTB9 cells (Figure 6c). In addition, miR-26b mimics markedly decreased Nox4 expression compared with miR-NC control (p < 0.01), while miR-26 inhibitor significantly increased Nox4 expression compared with inhibitor NC control (Figure 6d-e and supplementary Fig. 3). These results indicated that Nox4 was a target of miR-26b, and the effect of MI in bladder cancer was mediated by the miR-26b/Nox4 axis.

### *MI efficiently repressed the progression of bladder cancer* in vivo

**Figure 7.**
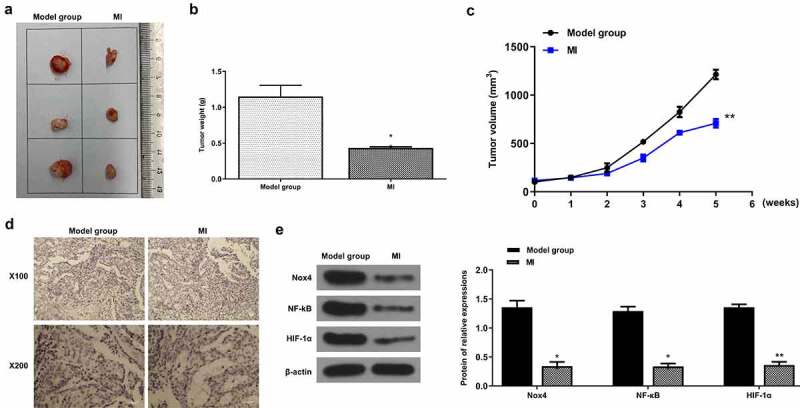
**MI efficiently repressed bladder cancer progression *in vivo***. (a) The representative images of xenograft tumors. (b) The tumor weight was evaluated on the 11^th^ day. (c) The tumor volume was evaluated every week for 5 weeks. (d) The Ki-67 staining of xenograft tumors. Magnification: × 100(up)/× 200(down). Scale bar = 100 μm. (e) Nox4, NF-κB, and HIF-1α protein levels in tumors from different groups were evaluated by Western blot. ** P < 0.01. Full-length blots/gels are presented in Supplementary Fig. 4.

Finally, the xenograft mouse model was established to determine the anticancer effect of MI on bladder cancer. The representative images of xenograft tumors from the model group and MI group are shown in Figure 7a. As expected, the tumor weight was significantly decreased in the MI group compared with the model group (p < 0.01) (Figure 7b). Meanwhile, the tumor volume was obviously decreased in a time-dependent manner in the MI group compared with the model group (p < 0.01) (Figure 7c). In addition, the Ki-67 staining assay showed that the number of positive cells in the MI group was markedly decreased compared with the model group (Figure 7d), suggesting that MI efficiently inhibited the proliferation of bladder cancer cells. Moreover, compared with the model group, the protein levels of Nox4 (p < 0.01), NF-κB (p < 0.01) and HIF-1α (p < 0.01) were significantly decreased compared with the model group (Figure 7e). These results demonstrated that MI efficiently repressed the progression of bladder cancer *in vivo*.

## Discussion

Due to its higher recurrence rate, bladder cancer brings physical agony and high therapy costs to the patients’ families and society [37,38]. Hence, the diagnosis and treatment of bladder cancer have drawn great attentions [39]. In the last decades, a series of natural or unnatural components have been identified and demonstrated certain anti-tumor activities in bladder cancer. For example, sulforaphane, a natural agent abundant in cruciferous vegetables, has been demonstrated to suppress the proliferation of non-muscle invasive bladder cancer cells via blocking HIF-1α-mediated glycolysis in hypoxia [40]. Inoue et al. reported a 5-aminolevulinic acid-mediated photodynamic therapy for bladder cancer and achieved some favorable clinical outcomes [41]. It was also reported that curcumin, a yellow substance of the polyphenols superfamily, attenuated bladder cancer progression partially by suppressing Sp-1 activity [42]. In addition, amygdalin, a natural compound, efficiently inhibited the growth of bladder cancer cells *in vitro* through diminishing cyclin A and cdk2 [43]. Despite many anti-tumor agents for bladder cancer, the identification of specific and efficient anti-bladder cancer agents is still urgent. In this study, we explored the effect of a hepatoprotective agent MI on bladder cancer and revealed that MI significantly inhibited the growth, invasion, and migration and induced apoptosis of bladder cancer cell lines HTB9 and BIU87 *in vitro*, suggesting a protective role of MI in bladder cancer development.

Recently, miR-26b has been a focus of interest for its role as a tumor suppressor in several cancer types. In colorectal cancer, miR-26b upregulation promotes chemo-sensitivity of cancer cells via targeting P-glycoprotein (Pgp) [44]. In esophageal squamous cancer, miR-26b inhibits cell proliferation by suppressing the c-MYC signaling pathway, suggesting that miR-26b might be a potential target to prevent and treat esophageal squamous cancer [45]. In lung cancer, miR-26b inhibits the invasion and migration of cancer cells by directly targeting hENT1 depending on the RhoA/ROCK-1 signaling pathway [46]. Although previous studies have revealed a crucial anti-tumor effect of miR-26 n in various cancers, its function in bladder cancer remains unclear. The present study revealed that MI significantly increases miR-26b expression in bladder cancer cell lines HTB9 and BIU87, suggesting that the protective effect of MI in bladder cancer is mediated by miR-26b.

To explore the specific mechanisms underlying the effect of MI on miR-26b in bladder cancer, Targetscan was applied to predict the potential targets of miR-26b. The prediction indicated that Nox4 might be a direct target of miR-26b. Meitzler et al. found that Nox4 expression is significantly upregulated in the carcinoma of bladder cancer patients compared with normal controls [47]. Recently, Nox4 has been revealed to play an important role in the invasion of bladder cancer cells [48]. One previous study reported that Nox 4 knockdown significantly inhibits the survival and induces apoptosis of bladder cancer cells [49]. These reports all confirmed a tumorigenic role of Nox4 in bladder cancer. To further verify the function of Nox4 *in vivo*, the xenograft mouse model was established to determine the anticancer effect of MI on bladder cancer. MI was intragastrically given to mice in the MI groups once per day for 5 weeks. Repeated experiments showed that MI at 0.1 mL/10 g body weight showed the best effects. To determine the interaction between miR-26b and Nox4, luciferase reporter assays were performed. The results showed that miR-26b mimics significantly decreased the relative luciferase activity of Nox4 WT vector, while miR-26b inhibitor significantly increased the relative luciferase activity of Nox4 WT vector. Meanwhile, miR-26b downregulation significantly increased Nox4 expression, and miR-26b upregulation decreased Nox4 expression. In addition, MI obviously decreased Nox4 expression both *in vitro* and *in vivo*. These results demonstrated that Nox4 is a direct target of miR-26, and the effect of MI on bladder cancer is mediated by the miR-26b/Nox4 axis.

Cell proliferation is one of the main features of solid tumor progression, and rapid tumor cell growth usually results in hypoxia because of the low oxygen environment [40]. A previous study indicated that one of the key factors regulating the response to hypoxia is the heterodimer hypoxia-inducible factor-1α (HIF-1α) [50]. A series of previous studies have demonstrated that HIF-1α can exacerbate bladder cancer progression, including promoting EMT process [51] and cell growth [52] and conferring the chemo-resistance to cisplatin of bladder cancer cells [53]. Our results showed that MI markedly decreases the expression of NF-κB and HIF-1α both *in vitro* and *in vivo*. These results suggest that MI induces miR-26b expression to downregulate Nox4 to reduce the expression of NF-κB and HIF-1α and subsequently inhibits proliferation and elevates apoptosis of bladder cancer cells. However, more investigation on the effects of MI on angiogenesis, hypoxia, and ROS production in bladder cancer needs to be performed in the subsequent experiments.

## Conclusion

MI efficiently inhibits bladder cancer progression both *in vitro* and *in vivo* by inhibiting the Nox4/NF-κB/HIF-1α signaling pathway by directly upregulating miR-26b expression, suggesting that MI might be a potential anticancer agent for bladder cancer.

## Supplementary Material

Supplemental Material

## Figures and Tables

**Table:** 

Magnesium Isoglycyrrhizinate	MI
wild type	WT
P-glycoprotein	Pgp
NAD(P)H oxidase 4	Nox4
non-small cell lung cancer	NSCLC.

## Data Availability

The analyzed data sets generated during the study are available from the corresponding author on reasonable request.

## Declarations

**Ethics approval and consent to participate**

The present study was approved by the Ethics Committee of The First Affiliated Hospital of Zhengzhou University. All procedures operated in keeping with the standards set out in the principles on ethical animal research outlined in the National Institutes of Health Laboratory Animal Care and Use Guidelines.
