# Supplementary figures and images for: Magnesium isoglycyrrhizinate suppresses bladder cancer progression by modulating the miR-26b/Nox4 axis
Source: Bioengineered. 2022 Mar 16;13(4):7986–99. doi: 10.1080/21655979.2022.2031677 (PMC9161837; doi:10.1080/21655979.2022.2031677)

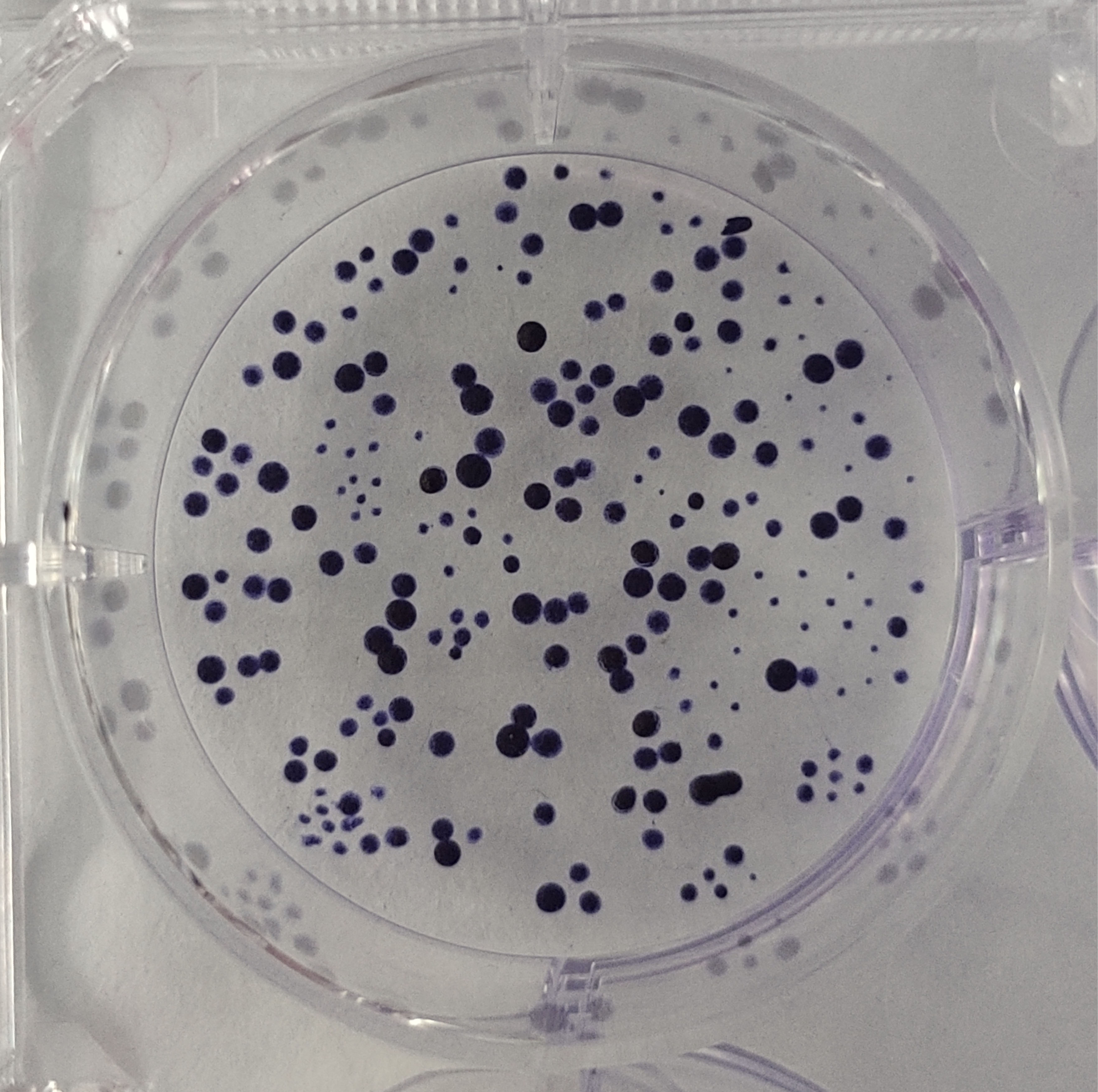

Supplement: Supplemental Material [file KBIE_A_2031677_SM8984.zip › supplementary/BIU87 0.jpg]

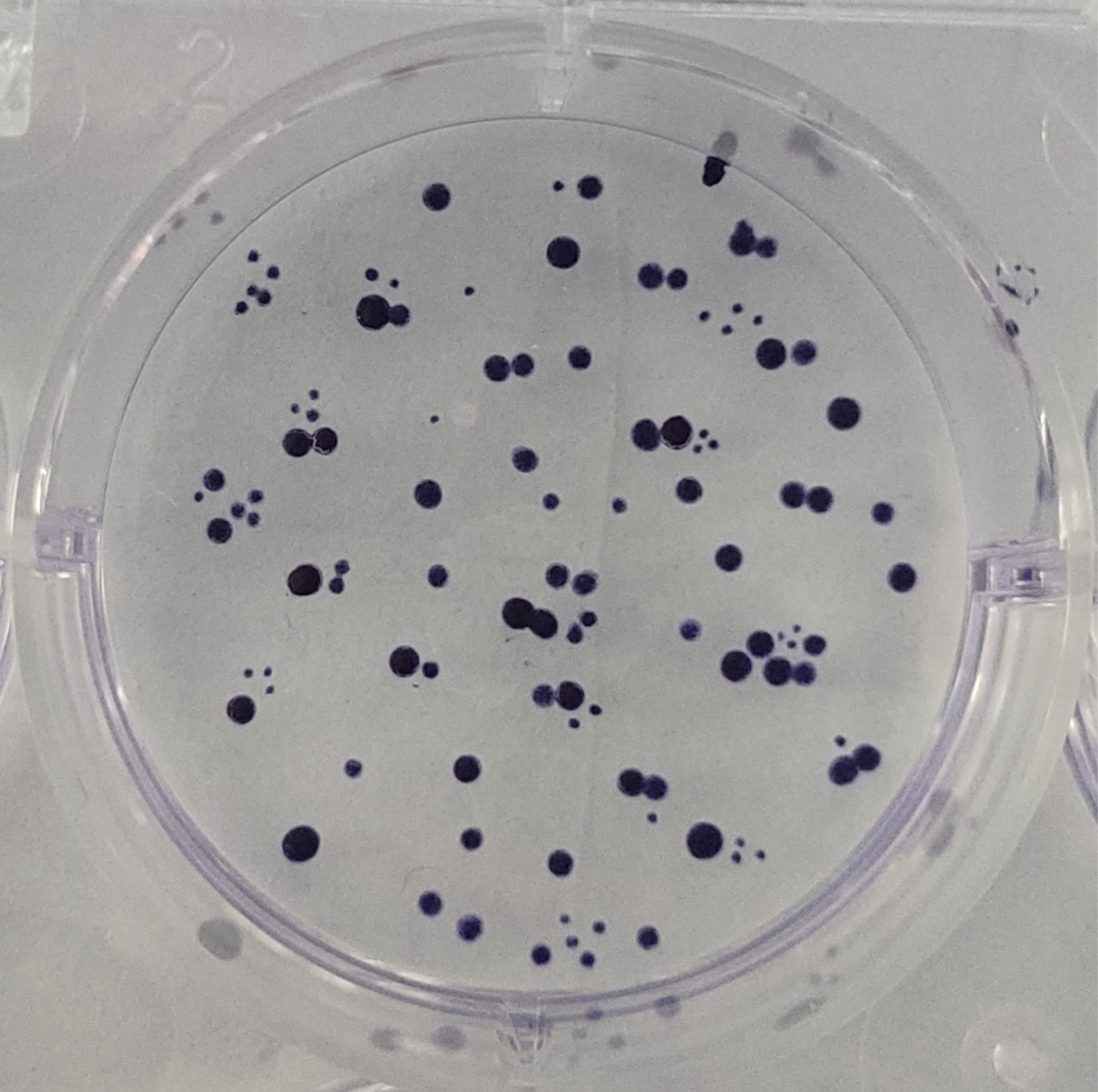

Supplement: Supplemental Material [file KBIE_A_2031677_SM8984.zip › supplementary/BIU87 2 85.jpg]

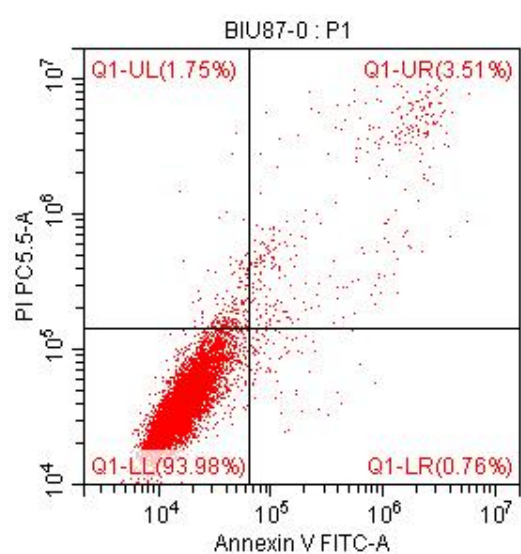

试管名称 : BIU87-0

样本ID :

| 群体           | %父群     |
|--------------|---------|
| ● All Events | 100.00% |
| ● P1         | 75.84%  |
| ● Q1-UR      | 3.51%   |
| ● Q1-UL      | 1.75%   |
| ● Q1-LL      | 93.98%  |
| ● Q1-LR      | 0.76%   |

Supplement: Supplemental Material [file KBIE_A_2031677_SM8984.zip › supplementary/BIU87_0.pdf]

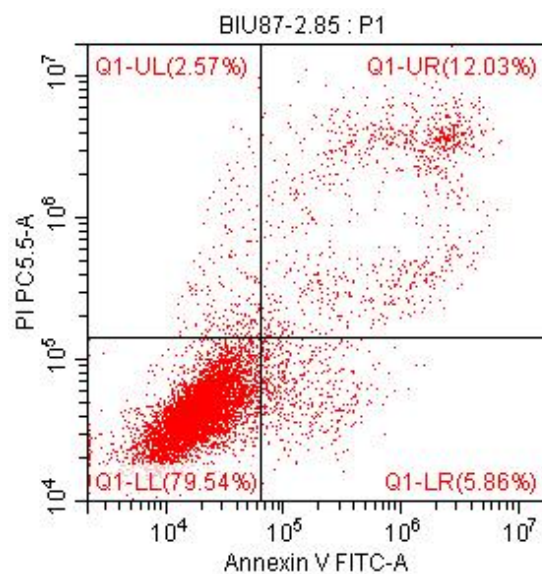

试管名称： BIU87-2.85

样本ID：

| 群体         | %父群     |
|------------|---------|
| All Events | 100.00% |
| P1         | 49.21%  |
| Q1-UR      | 12.03%  |
| Q1-UL      | 2.57%   |
| Q1-LL      | 79.54%  |
| Q1-LR      | 5.86%   |

Supplement: Supplemental Material [file KBIE_A_2031677_SM8984.zip › supplementary/BIU87_2 85.pdf]

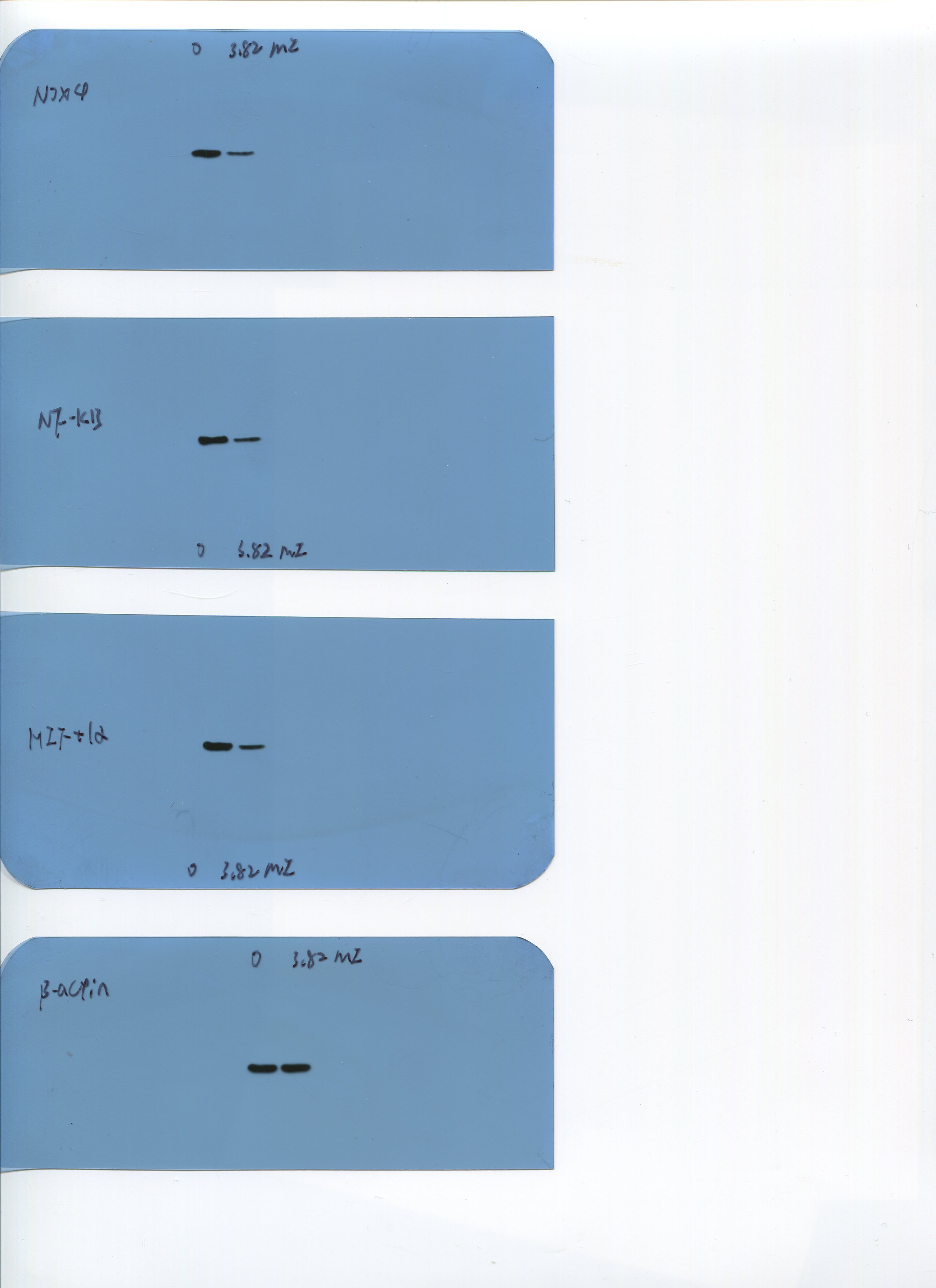

Supplement: Supplemental Material [file KBIE_A_2031677_SM8984.zip › supplementary/Figure 5B Nox4 NF__B HIF_1_ __actin.jpg]

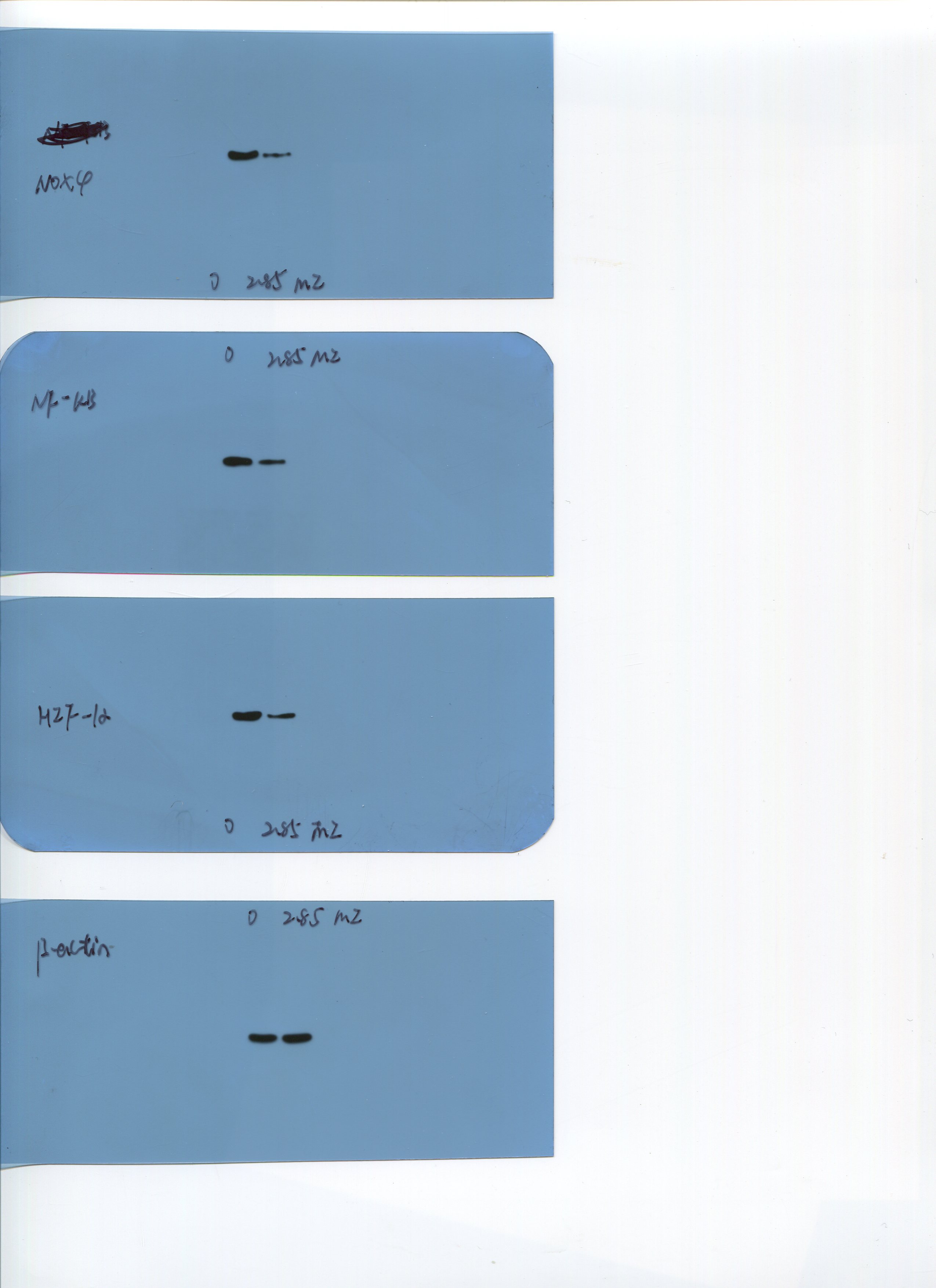

Supplement: Supplemental Material [file KBIE_A_2031677_SM8984.zip › supplementary/Figure 5C Nox4 NF__B HIF_1_ __actin.jpg]

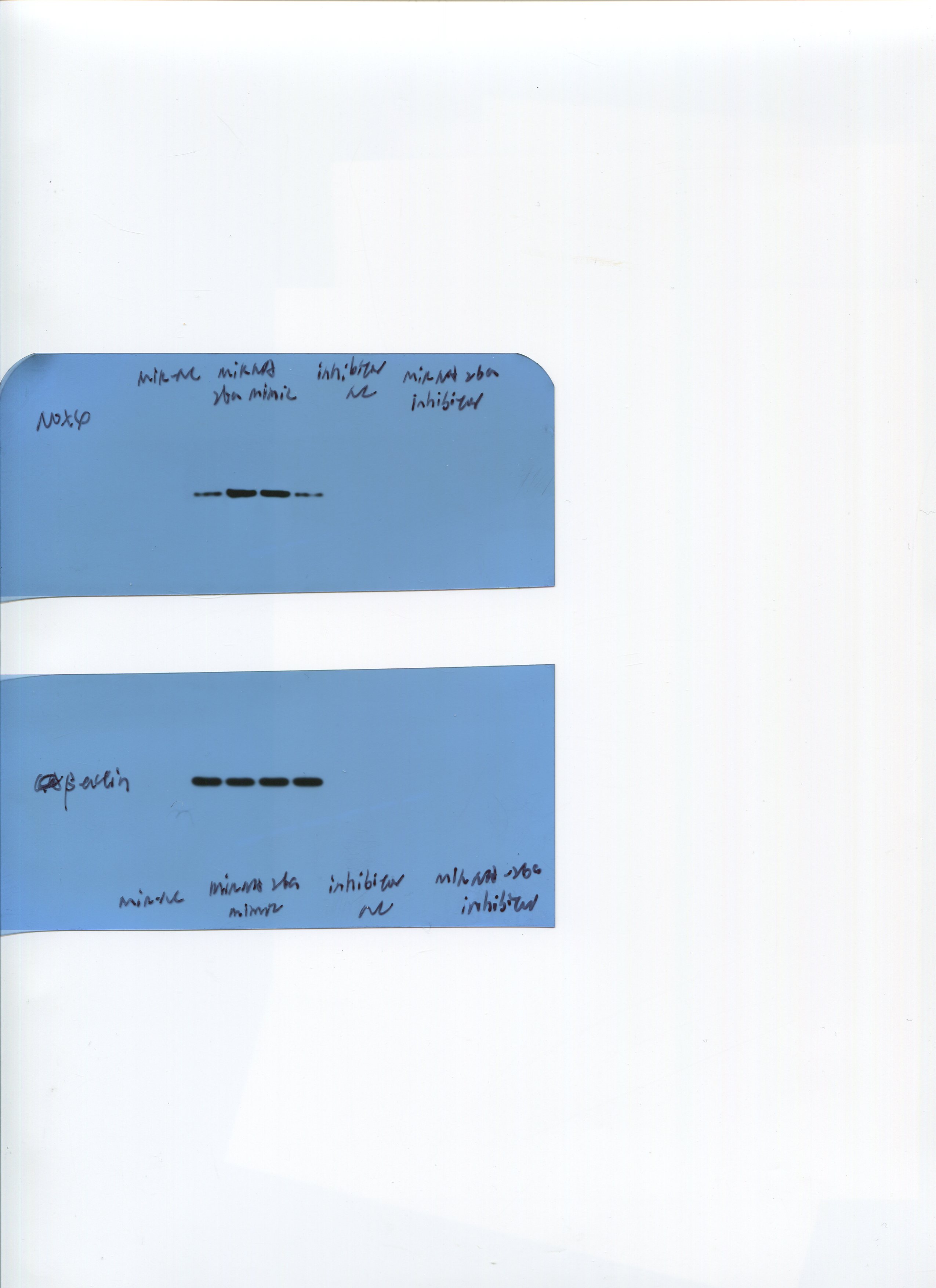

Supplement: Supplemental Material [file KBIE_A_2031677_SM8984.zip › supplementary/Figure 6D Nox4 __actin.jpg]

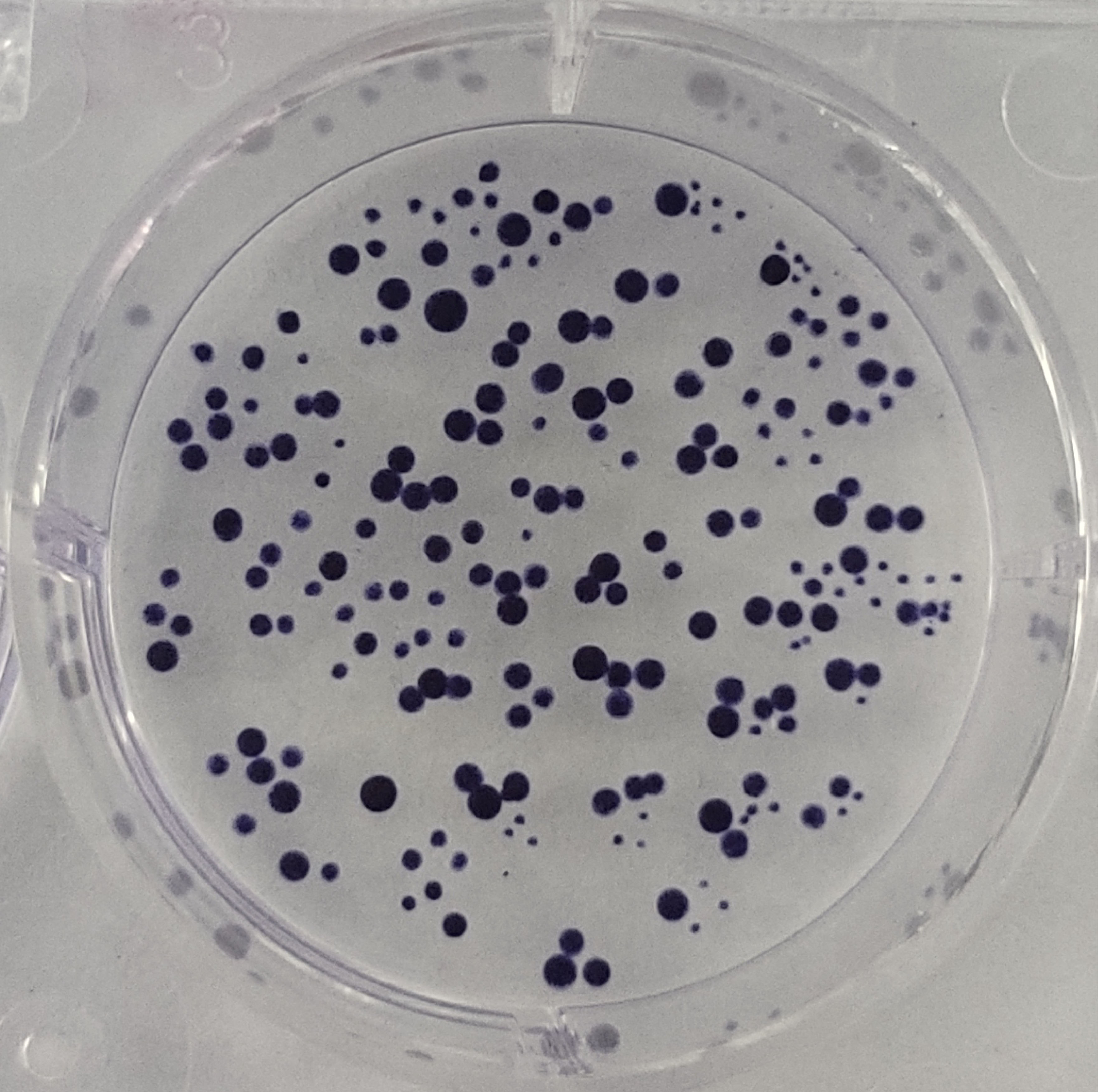

Supplement: Supplemental Material [file KBIE_A_2031677_SM8984.zip › supplementary/HTB29 0.jpg]

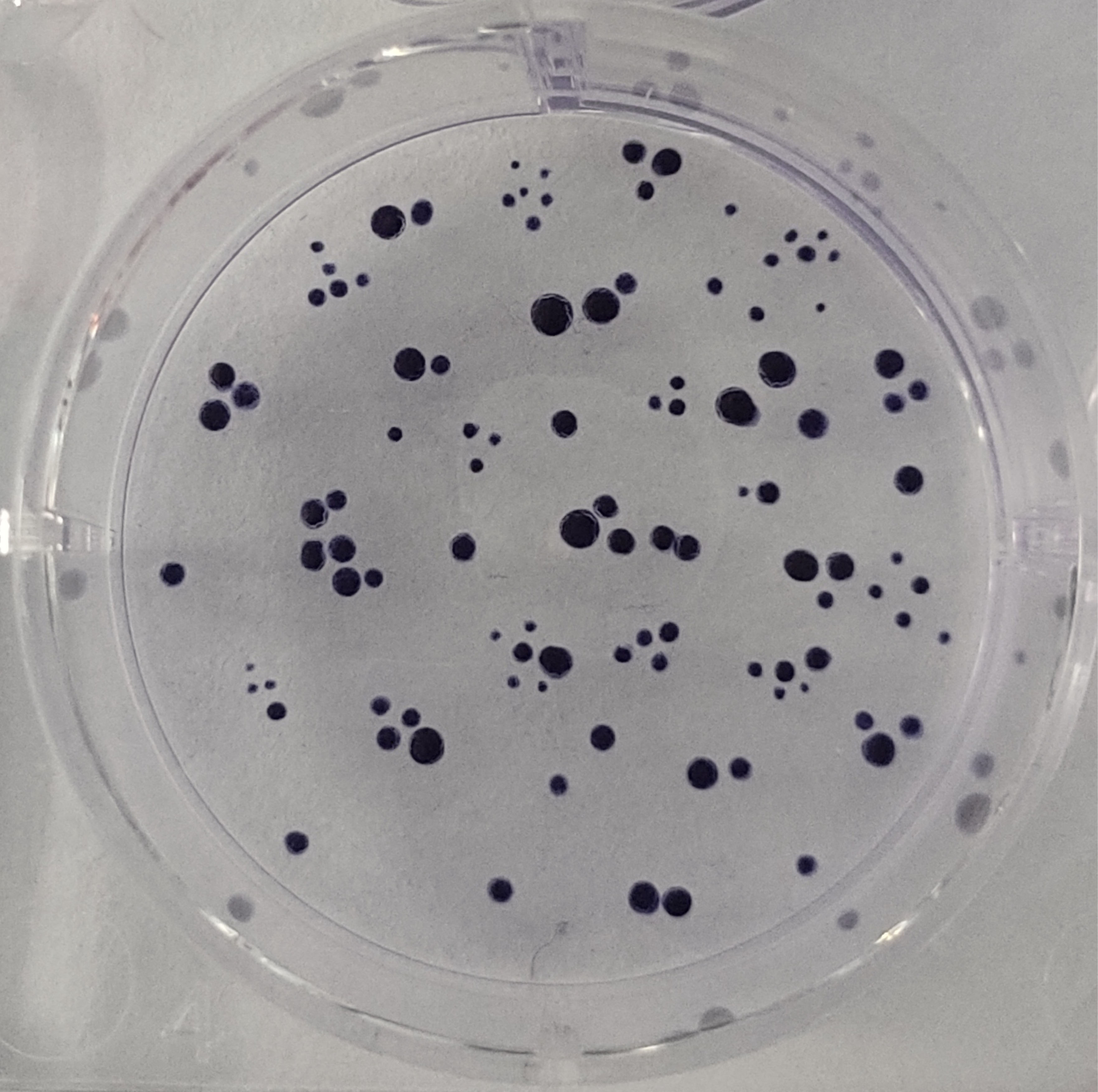

Supplement: Supplemental Material [file KBIE_A_2031677_SM8984.zip › supplementary/HTB29 3 82_01.jpg]

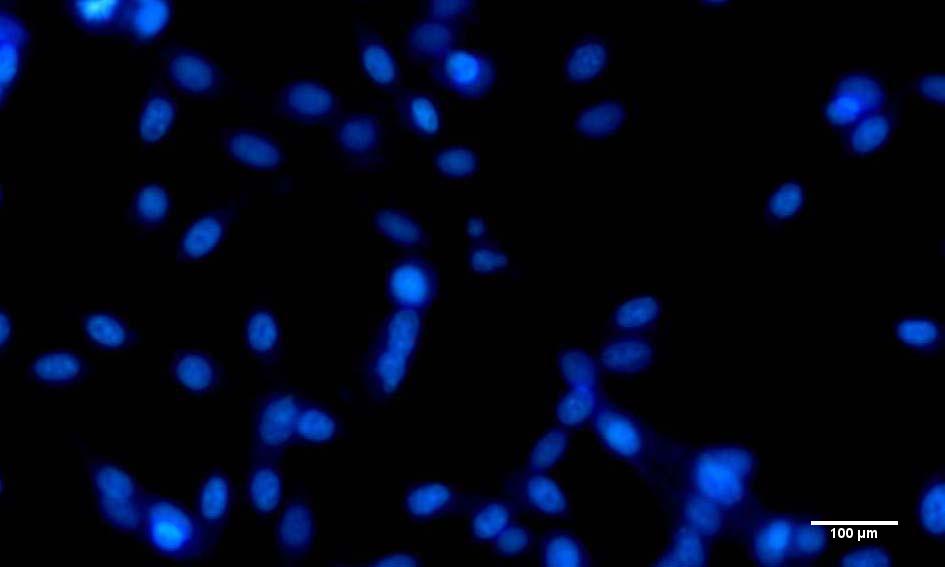

Supplement: Supplemental Material [file KBIE_A_2031677_SM8984.zip › supplementary/HTB9 0.jpg]

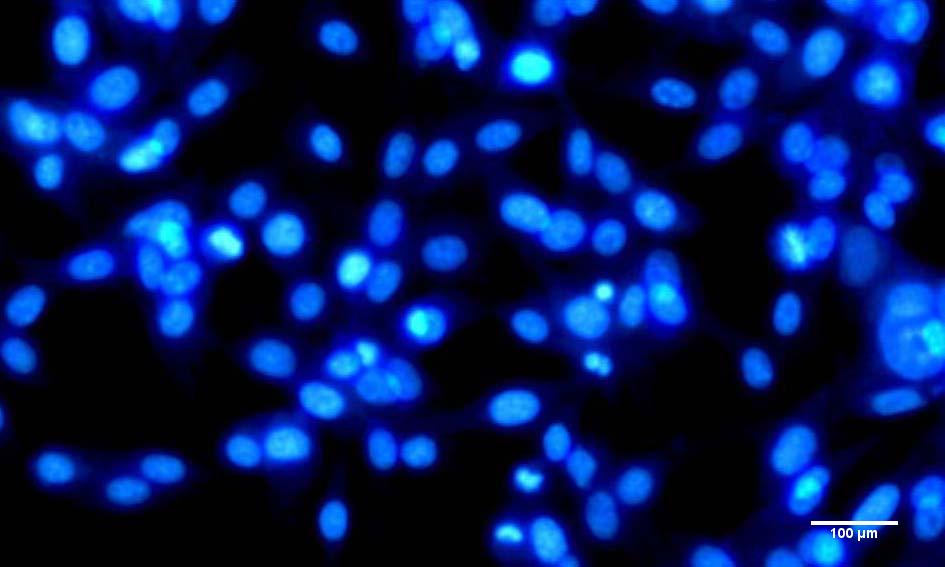

Supplement: Supplemental Material [file KBIE_A_2031677_SM8984.zip › supplementary/HTB9 3 82.jpg]

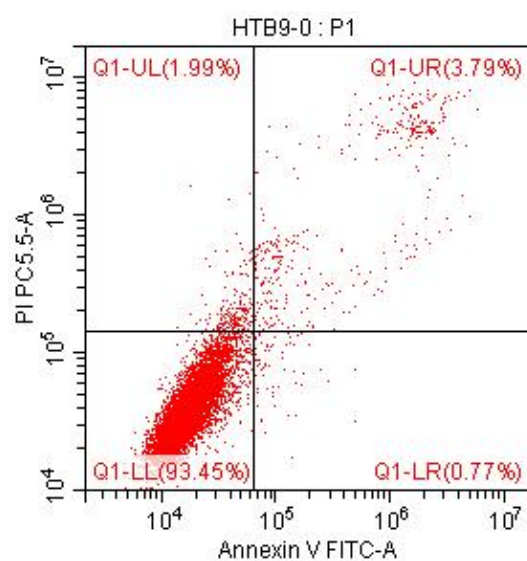

试管名称： HTB9-0

样本ID：

| 群体           | %父群     |
|--------------|---------|
| ● All Events | 100.00% |
| ● P1         | 74.55%  |
| ● Q1-UR      | 3.79%   |
| ● Q1-UL      | 1.99%   |
| ● Q1-LL      | 93.45%  |
| ● Q1-LR      | 0.77%   |

Supplement: Supplemental Material [file KBIE_A_2031677_SM8984.zip › supplementary/HTB9_0.pdf]

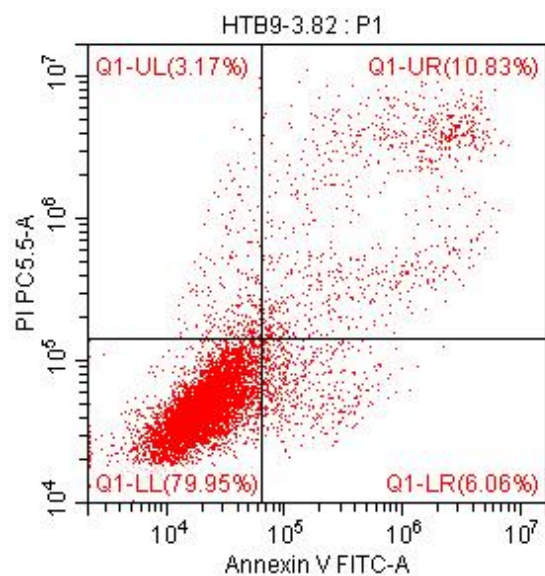

试管名称： HTB9-3.82

样本ID：

| 群体           | %父群     |
|--------------|---------|
| ● All Events | 100.00% |
| ● P1         | 50.37%  |
| ● Q1-UR      | 10.83%  |
| ● Q1-UL      | 3.17%   |
| ● Q1-LL      | 79.95%  |
| ● Q1-LR      | 6.06%   |

Supplement: Supplemental Material [file KBIE_A_2031677_SM8984.zip › supplementary/HTB9_3 82.pdf]
