# Supplementary material for: Magnesium isoglycyrrhizinate suppresses bladder cancer progression by modulating the miR-26b/Nox4 axis
Source: Bioengineered. 2022 Mar 16;13(4):7986–99. doi: 10.1080/21655979.2022.2031677 (PMC9161837; doi:10.1080/21655979.2022.2031677)
Supplement: Supplemental Material [file KBIE_A_2031677_SM8984.zip › supplementary/Supplementary Figure 1_4.doc]

Supplementary Fig. 1 Full-length blots/gels of Figure 5B.

Supplementary Fig. 2 Full-length blots/gels of Figure 5C.

Supplementary Fig. 3 Full-length blots/gels of Figure 6E.

Supplementary Fig. 4 Full-length blots/gels of Figure 7F.
